# Neurexin-3a IgG-mediated autoimmune encephalitis: a case report and literature review

**DOI:** 10.3389/fimmu.2025.1542578

**Published:** 2025-05-21

**Authors:** Pin Wang, Lin Sun, Yan Wei, Ling Cheng, Haoyang Cheng, Peiyu Shi, Yingying Xu

**Affiliations:** ^1^ Department of Neurology Medicine, The Second Hospital of Shandong University, Cheeloo College of Medicine of Shandong University, Shandong University, Jinan, China; ^2^ The Second Hospital of Shandong University, The Second Clinical College of Shandong University, Shandong University, Jinan, China; ^3^ State Key Laboratory of Neurology and Oncology Drug Development, Jiangsu Simcere Diagnostics Co., Ltd., Nanjing, China

**Keywords:** autoimmune encephalitis, neurexin-3a IgG-mediated autoimmune encephalitis, cell-based assay, tissue-based assay, immunotherapy

## Abstract

We summarized the clinical manifestations, auxiliary examinations, treatment, and prognostic characteristics of a patient with neurexin-3a IgG-mediated autoimmune encephalitis. On March 2, 2024, a 43-year-old male patient was admitted to the Second Hospital of Shandong University and had prodromal symptoms of infection before the onset of encephalitis. The main manifestations were episodic loss of consciousness, eyes turned upward to the right, clenched teeth, bleeding from tongue bite, and limb twitching. Imaging results showed that the left frontal lobe was characterized by a patchy, slightly longer T1 and T2 signal foci, with a slightly higher signal in the pressurized water image. The CSF virus test was normal; both the serum and CSF were positive for neurexin-3a antibodies using CBA, which were confirmed by TBA. The patient’s symptoms improved after glucocorticosteroid therapy. Neurexin-3a IgG-mediated autoimmune encephalitis is a new type of autoimmune encephalitis, and suspicion of associated disease requires further testing for neurexin-3a IgG for a definitive diagnosis.

## 1 Introduction

Autoimmune encephalitis (AE) is an inflammatory disease of the brain parenchyma with clear triggers of infection and tumor. It is mediated by autoimmune mechanisms and pathologically characterized by inflammatory lesions in the gray matter and brain neurons, with some involvement of white matter. The clinical manifestations caused by AE are complex and varied. Patients present with an acute or subacute onset of disease, and AE is mainly characterized by near-memory deficits, psychiatric-behavioral abnormalities, seizures, and altered consciousness (1). Antibodies to neuronal intracellular antigens typically associate with paraneoplastic neurological syndromes and poor prognosis, whereas antibodies to synaptic/neuronal cell surface antigens characterize many AE subtypes that associate with tumors less frequently, and that are often immunotherapy-responsive (2). With the development of antibody detection technology and the discovery of neurological antibodies, knowledge of AE has become more extensive, and the differences in the clinical features and immunotherapy of various antibody-mediated AE have been reported. In 2016, Gresa-Arribas et al. identified a novel antibody against neurexin-3a in five patients with AE (3); however, reports on this antibody-mediated AE are rare. Herein, we described a case of neurexin-3a antibody-mediated AE. This report contributes to a better understanding of this type of disease, paving the way for improved diagnosis and treatment.

## 2 Case presentation

### 2.1 Clinical information

On March 2, 2024, a 43-year-old man was admitted to our hospital due to “episodic loss of consciousness for more than a month.” The patient had a sore throat, runny nose, cough, small amount of sputum, no fever, and above-mentioned symptoms improved with cold medication. Three days later, he lost consciousness, fell to the ground, and had both eyes fixed to the upper right, teeth clenching, and limb convulsions, which lasted approximately 30 s and then got better, with no vomiting or incontinence. Eight days prior to admission to the local hospital, the patient had a recurrence of the above-mentioned seizures with tongue biting, which lasted approximately 1 minute before improving. He was brought to the local hospital, and a thyroid ultrasound was performed, showing diffuse lesions in the thyroid parenchyma and the possibility of Hashimoto’s thyroiditis. Chest computed tomography(CT) results suggested inflammation in the upper lobe of the right lung, but those of the epigastric region, pelvis, and cranial brain, as well as magnetic resonance imaging (MRI), showed no obvious abnormalities. The patient underwent tongue suture placement and administered sodium valproate 0.2g tid, acyclovir 500 mg q8h, and piperacillin 4.5g q8h orally. Because the patient continued to have several seizures lasting a few seconds since being discharged from the local hospital, he was admitted to the emergency department of the same hospital with “cause of encephalitis to be investigated.” Since disease onset, the patient was on a general diet and had poor sleep quality, no abnormalities in urination and defecation, and no recent significant changes in weight. Physical examination on emergency admission showed a body temperature of 36.8°C, pulse of 79 beats/min, respiration of 17 beats/min, and blood pressure of 120/77 mmHg. The patient’s consciousness was clear, and he was slightly unresponsive. He also had a decline in gross near-memory, computation, and orientation, with a total score of 15 on the Brief Mental Status Examination Scale (MMSE), suggesting moderate cognitive deficits. His Montreal Cognitive Assessment Scale (MOCA) score was 11, suggesting cognitive dysfunction. The rest of the neurological examination showed no abnormality.

### 2.2 Complementary examination

**Figure 1:**
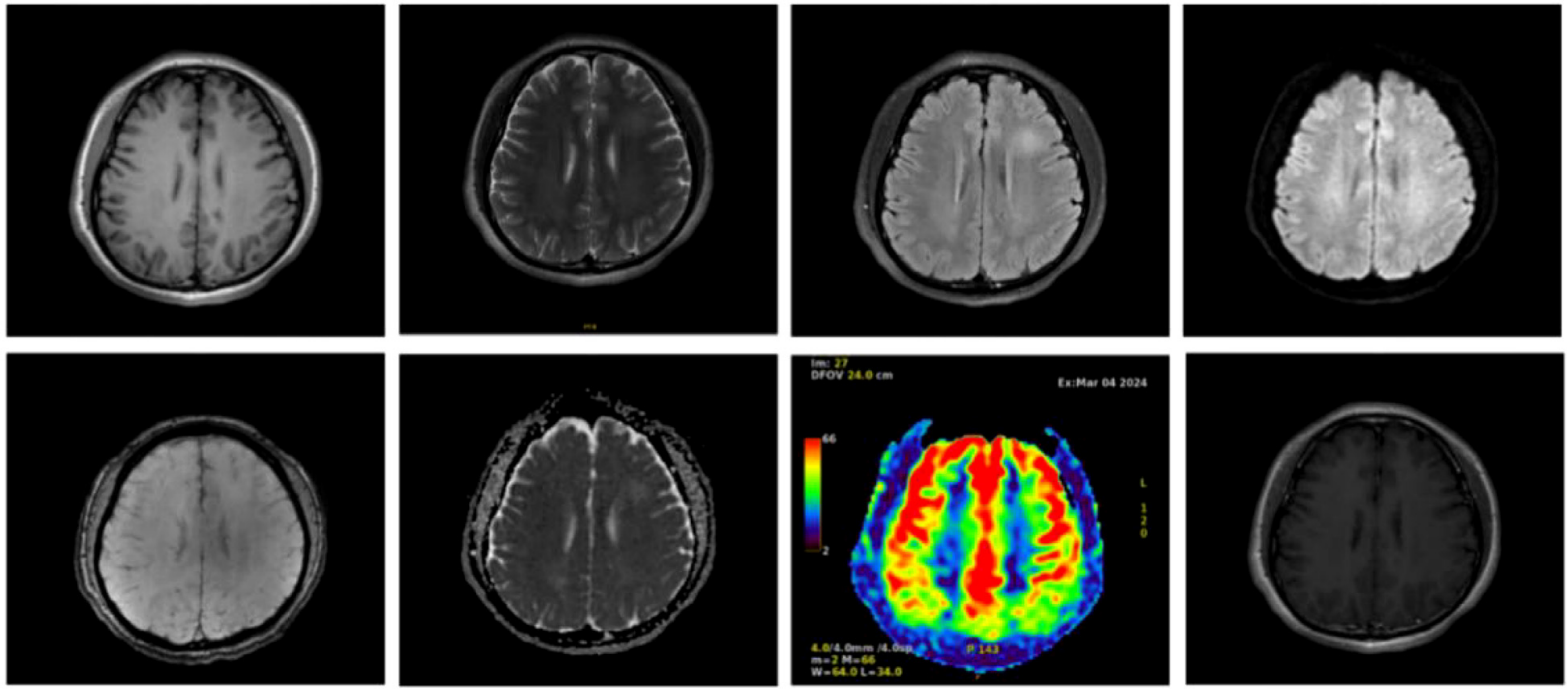
Cranial magnetic resonance imaging of the patient with autoimmune encephalitis showing a frontal lobe lesion, no abnormal low-signal foci in SWAN, and no significant abnormalities in blood flow in the lesion area in ASL.

During electroencephalography(EEG) monitoring, each lead showed paroxysmal medium-to-high amplitude 2–3 Hz δ waves, with a few 4–7 Hz theta waves. MRI showed small patchy foci of slightly longer T1 and T2 signals in the left frontal lobe, with a slightly higher signal in the pressurized water image. Diffusion-weighted imaging(DWI) was unremarkable (**Figure 1**). Abnormal low-signal foci were not detected in T2 star-weighted angiography(SWAN), and any abnormality in the blood flow in the lesion area was not detected in arterial spin labeling(ASL). A complete blood count showed a leukocyte count of 2.34 × 10^9^/L, monocyte proportion of 14.5%, neutrophil count of 1.51 × 10^9^/L, lymphocyte count of 0.47 × 10^9^/L, and calcitonin of 0.058 ng/ml.

Rheumatological parameters showed a positive ANA test of nuclear antibodies with titer 1:3200, with titer 1:3200 nuclear granulomatous and titer 1:320 cytoplasmic granulomatous karyotypes. In addition, SSA/Ro52 and Sm antibodies were positive. No significant abnormalities in thyroid function, RF, ASO, antineutrophil antibodies, lupus anticoagulant, infectious disease series, liver and renal function glycolipid biochemistry, folic acid and vitamins, tumor markers, and T-SPOT were detected. The cerebrospinal fluid (CSF) was colorless and clear and had 15/mm^3^ leukocyte count and 85% lymphocyte proportion, the intracranial pressure was 160 mmH_2_O, the cytology was lymphocytic, and the tryptophan test was negative. CSF biochemistry suggested normal protein, sugar, and chloride levels.

**Figure 2:**
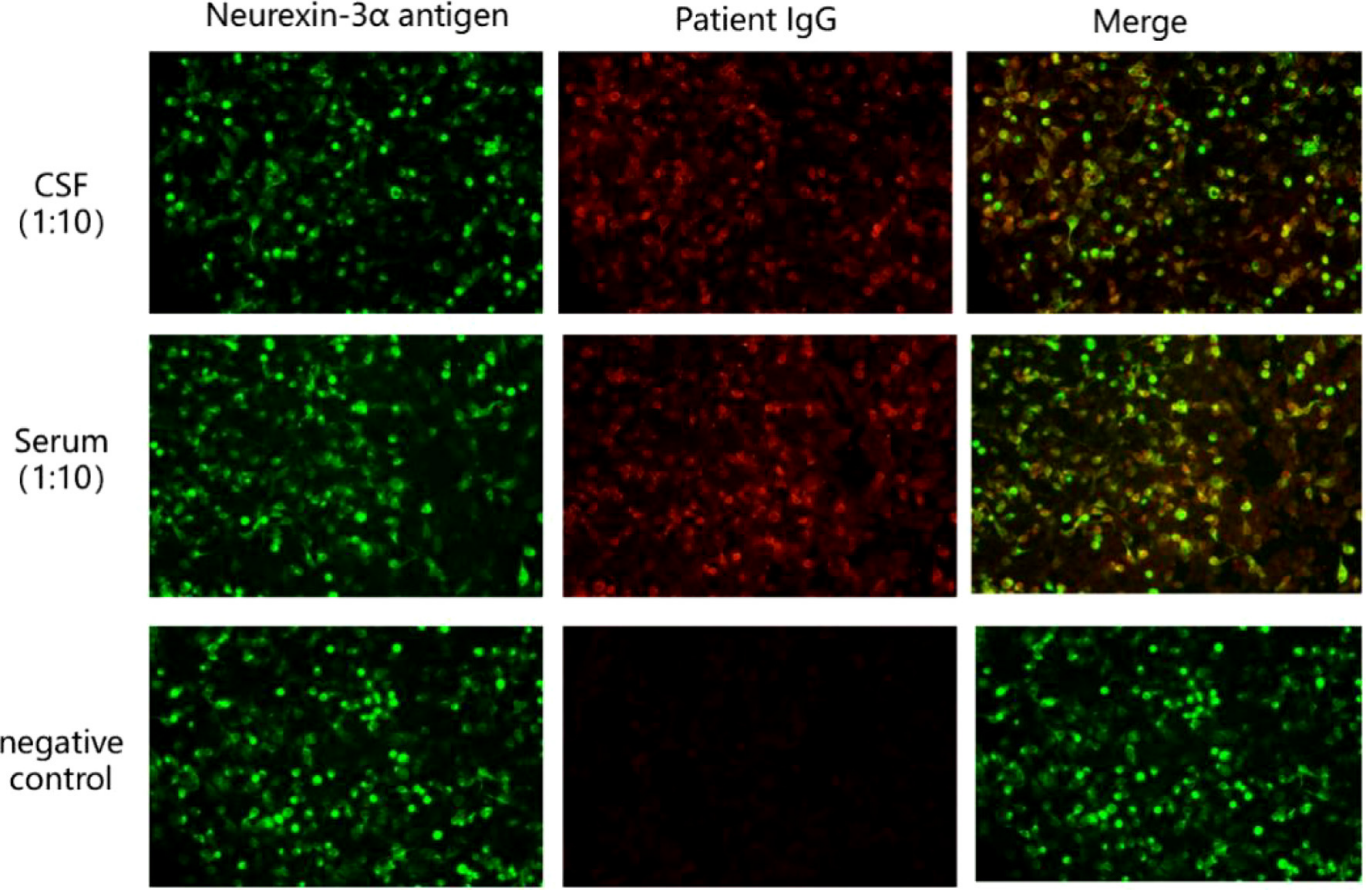
Presence of neurexin-3a antibody was confirmed by cell-based assays in the patient’s serum and CSF. Neurexin-3a-transfected HEK293T cells are shown in green, while Neurexin-3a IgG are in red.

**Figure 3:**
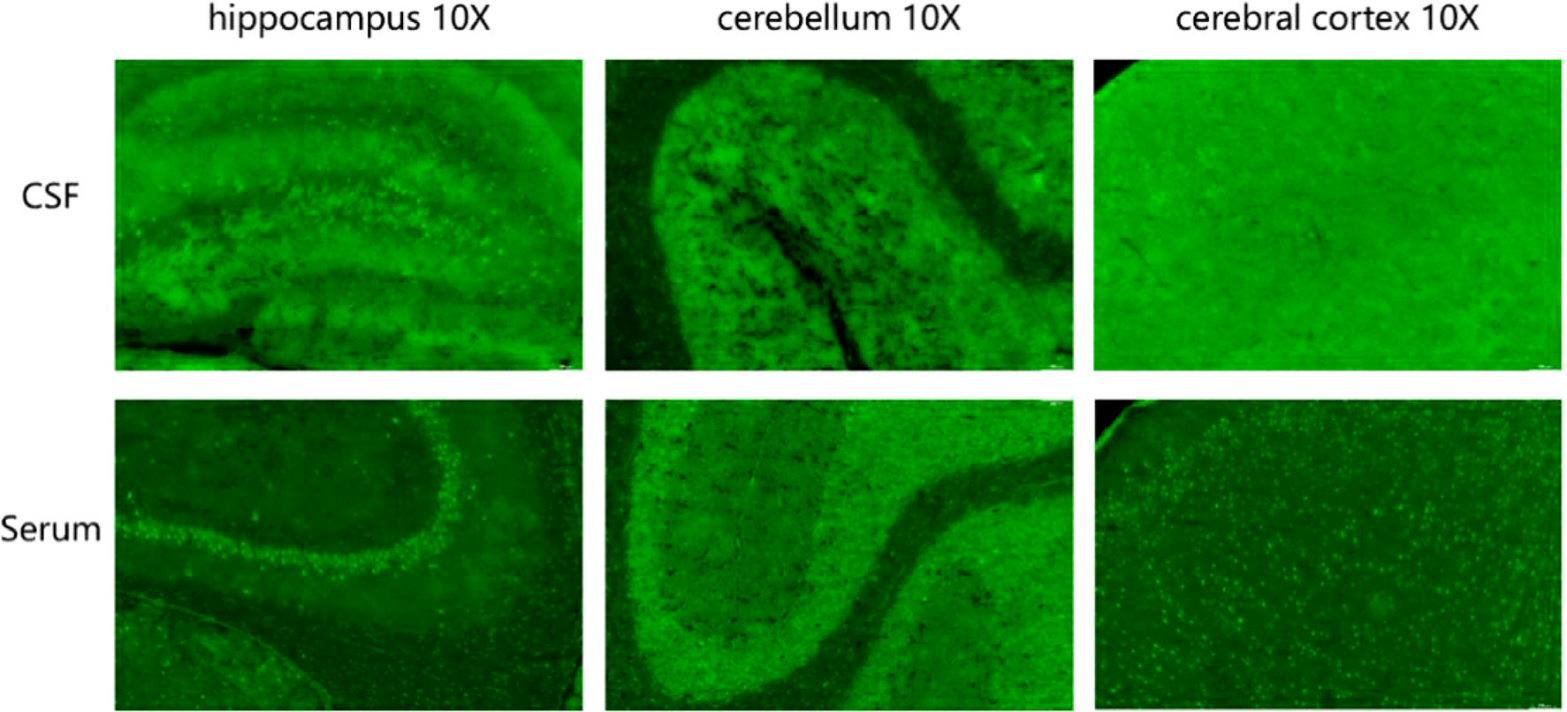
Tissue-based assay (TBA) showing the presence of immunoreactivity of cerebrospinal fluid and serum antibodies in the rat hippocampus, cerebellum, and cerebral cortex.

The results of ink stain, Gram stain, Alisinolan stain, cryptococcal pod antigen stain, and antacid stain were negative. The CSF was also negative for viral antibodies. Neural antibodies associated with AE and paraneoplastic syndrome, including neurexin-3a, NMDAR, LGI1, CASPR2, GABA_B_R, AMPAR1, AMPAR2, DPPX, GAD65, mGluR5, GlyR, D2R, IgLON5, Hu, Yo, and Ri, in the serum and CSF were tested using cell-based assays (CBA) with immunofluorescence double staining. The screening titration was started from 1:10 and 1:1 for the serum and CSF samples, respectively, followed by a 10-fold dilution for the positive sample. The antibodies in the serum and CSF were further confirmed by tissue-based assay (TBA) using rat brain and kidney tissues (1:100). All tests were performed in Jiangsu Simcere Diagnostic Laboratory (Nanjing, China). CBA results suggested that both the blood and CSF were 1:10 positive for neurexin-3a antibody (**Figure 2**); meanwhile, TBA results indicated that the hippocampus, cerebellum, and cerebral cortex showed neuronal membrane antibody-positive fluorescence (**Figure 3**).

### 2.3 Treatment and follow-up

The patient was treated with glucocorticosteroids (1000 mg) and antiepileptic treatment. No further seizures occurred during hospitalization, and cognitive impairment was significantly improved at discharge. After discharge, the patient was given oral prednisone 60 mg per day and sodium valproate 500 mg bid. During follow-up 1 month later, no seizures were reported, and cognitive impairment improved. His MMSE score improved to 20 and his MOCA score was 15.

## 3 Discussion

Since the first report of N-methyl-D-aspartate receptor (NMDAR) encephalitis by Dalmau et al. in 2007 (4), other antibodies such as γ-aminobutyric acid type B receptors (GABABR) and Leucine-rich glioma inactivated 1 (LGI1) that can cause AE have been reported. Synaptic proteins are among the most diverse families of proteins in the mammalian nervous system, with more than 1000 isoforms (5). They are highly expressed in the presynaptic membrane and play important roles in synaptic cell adhesion and neurotransmitter secretion (6). The pathogenesis of neurexin-3a antibody-associated encephalitis is hypothesized as follows: neurexin-3a antibodies suppress neuronal neurexin-3a expression and decrease the total number of synapses, thus affecting synapse development (3). In addition, neurexin-3a antibodies can disrupt the balance between inhibitory and excitatory synapses in different regions of the CNS regulated by neurexin-3a (7).

The clinical features of neurexin-3a antibody-mediated AE are preceded by infection-like prodromal symptoms, which may be accompanied by headache and gastrointestinal symptoms and followed by seizures, memory loss, confusion, loss of consciousness, central hypoventilation, behavioral abnormalities, and speech disorders (8). Of the 15 patients (including the case patient) with neurexin-3a antibody-mediated AE reported thus far, 8 were women, and 7 were men. Among them, 10 had symptoms of antecedent infection (e.g., fever, headache, high temperature, nausea, or diarrhea); 13, decreased level of consciousness and seizures; 4, central hypoventilation; and 2, severe hallucinations and hallucinations (**Table 1**). In this case report, the patient had prodromal symptoms of infection, including sore throat, runny nose, cough, coughing up a small amount of sputum, and no fever. These were followed by episodic loss of consciousness, which appeared as eyes fixed to the upper right, teeth clenching, bleeding from tongue bite, and limb convulsions, lasting for 1 minute. A history of antecedent infections can contribute to the disruption of the blood–brain barrier due to infectious inflammation, and antibodies can enter the CNS through the damaged blood–brain barrier, leading to the development of immune-inflammation in the CNS. Because neurexin-3a antibody-mediated encephalitis can be preceded by symptoms of prodromal infection, some scholars believe that neurexin-3a antibody-mediated encephalitis is post-infectious encephalitis (18).

**Table 1:** Main clinical data of reported patients with neurexin-3a antibody-mediated encephalitis (n = 15).

Patient	Sex	Age (years)	Prodromal symptoms	Main clinical manifestations	MRI	CSF findings	Treatment	Prognosis	Other characteristics
1 (3)	Female	23	Headache	Confusion, drowsiness, memory impairment, and suspected central hypoventilation	Normal	First: normal Second: white blood cell count 20	Corticosteroids	Followed up after 36 months: partial recovery of symptoms, residual memory deficits, anxiety, nocturnal bilevel positive airway pressure ventilation	Complicated with leukopenia, transient thrombocytopenia
2 (3)	Male	23	Headache, fever	Blurred consciousness, extreme agitation, generalized spasms, myoclonus, coma	Normal	White blood cell count: 31	Corticosteroids	No effect of treatment; died on day 17	Accompanied by thrombocytopenia, leukopenia, antinuclear antibodies of 1:60; autopsy suggested cerebral edema and brain herniation
3 (3)	Female	50	Headache, fever, vomiting, diarrhea	Abnormal behavior, seizures, decreased level of consciousness, mild orofacial dyskinesia, central hypopnea	Abnormal signal of FLAIR/T2 and DWI in the medial temporal lobe	Increased IgG index	corticosteroids, cyclophosphamide	Followed up after 4 months: partial recovery of symptoms, residual cognitive impairment, and refractory epilepsy	History of systemic lupus erythematosus and Raynaud’s disease
4 (3)	Female	44	Headache, fever	Confusion, agitation, seizures, incoherent speech, mild orofacial dyskinesia	Normal	10 white blood cells	Corticosteroids, immunoglobulin	Died after the 67th day of septicemia	Antinuclear antibody of 1:1 280; autopsy showed mild subarachnoid hemorrhage, and lung showed cytomegalovirus infection
5 (3)	Female	44	Nausea, diarrhea	Confusion, memory impairment, seizures	Normal	24 white blood cells	Corticosteroids	Followed up after 2 months: significant recovery of symptoms, residual mild cognitive impairment (still improving)	With chronic arthralgia, positive thyroglobulin antibodies
6 (9)	Male	57	Fever, fatigue, dizziness, muscle pain	Apathy, drowsiness, interrupted sleep-wake cycle, confusion and behavioral changes	Slightly high signal in the caudate nucleus-inner capsule gap	159 cell counts (91% monocytes)	Corticosteroids	Complete relief of symptoms after 15 days	Diagnosis of Plasmodium falciparum infection prior to onset of disease
7 (10)	Male	58	None	Seizures, memory loss, depressed mood, psychomotor retardation	Abnormal signal in the left temporal lobe	Elevated levels of phosphorylated tau protein (70 pg/mL)	Corticosteroids	No apparent change	Positive Yo and SOX1 antibodies; 18F-FDG-PET/CT showed marked hypometabolism in the bilateral prefrontal and parietal lobes
8 (11)	Female	59	Non-specific prodromal symptoms, not detailed	Blurred consciousness, decreased concentration, disorientation, seizures	Increased volume of the left hippocampus, T2 high signal	Mild cellular proliferation	Corticosteroids, plasma exchange, rituximab	Complete remission on day 29	History of rheumatoid arthritis, Hashimoto’s thyroiditis
9 (12)	Male	56	Fever, cough	Memory loss, seizures, myoclonic seizures, involuntary movements, dystonia, autonomic abnormalities	FLAIR high signal in the bilateral temporal lobe, hippocampus, and insula	Normal at 2 times	Corticosteroids, immunoglobulin	Died 10 days later	NA
10 (13)	Female	21	NA	Progressive motor retardation, apathy and hypotonia, unsteady walking, trembling of limbs	Slight dilatation of the ventricular system, atrophic changes in the brain with widening of the sulcal pools	Leukocytes 14×10^6^/L, protein level 498.50 mg/L, glucose 4.56 mmol/L	Corticosteroids, immunoglobulins, azathioprine	Symptoms improved after 16 days, relapsed on self-discontinuation, improved on retreatment	Psychological stimuli prior to onset of illness
11 (14)	Female	42	Headache and fever after yellow fever vaccination	Seizures, psychotic symptoms, disorders of consciousness, hallucinations, delusions of victimization, and agitation	NA	Leukocytes 15/mm3, Normal protein	Corticosteroids, immunoglobulins, azathioprine	Walking with unilateral support after three months, occasional mild paranoia of victimization, otherwise functioning normally	Positive antibodies to ANA, ds-DNA, and SSA; history of arthritis, confirmed diagnosis of SLE
12 (15)	Female	74	NA	Bilateral lower extremity weakness, cognitive impairment	Hypometabolism in the prefrontal and parietal lobes, hypermetabolism in the medial temporal lobe	NA	Plasma exchange, immunoglobulins, corticosteroids	Immunotherapy was ineffective and eventually underwent stereotactic needle brain biopsy of a right frontal lobe lesion	Pathology of the posterior frontal lobe showing diffuse large B-cell lymphoma
13 (16)	Male	54	NA	Recurrent generalized tonic–clonic seizures, impaired consciousness, psychotic symptoms (such as hallucinations), cognitive dysfunction, and a significant decrease in sleep time	Cerebral edema and meningitis, with major consideration of contrast-induced encephalopathy	Increased white blood cells and protein	Corticosteroids, MMF	Recovered	Postcarotid stenting
14 (17)	Male	18	Headache, fever	Delirium of consciousness, new-onset refractory status epilepticus (NORSE)	Normal	Increased cells, lymphocyte predominance, elevated proteins	Plasma exchange, immunoglobulins, corticosteroids	Died 19 days after admission	Lymphopenia, mild thrombocytopenia, and elevated transaminases
15 this case	Male	43	Sore throat, runny nose, and cough	Loss of consciousness, seizures	Lesion in the frontal lobe	Leukocytes 15/mm3, lymphocytes 85%	Corticosteroids	Recovered	Antinuclear antibodies of titer 1:3200 (titer 1:3200 nuclear granular, titer 1:320 cytoplasmic granular), positive SSA/Ro52, and Sm antibodies

The cranial MRI findings of patients with neurexin-3a antibody-mediated encephalitis may be normal or abnormal. Among the 15 patients with neurexin-3a antibody-mediated encephalitis reported thus far, 4 had normal cranial MRI manifestations, 9 had abnormal signals in different brain regions, and 2 lacked detailed information (**Table 1**). Abnormal MRI signals were found in the brain of some cases, such as in the temporal lobe, hippocampus, medial temporal lobe or caudate-capsule-lenticulate regions. In our patient, the MRI scan showed a patchy, slightly longer T1 and slightly longer T2 signal foci in the left frontal lobe, with a slightly higher signal in the pressurized water image. All patients with neurexin-3a antibody-mediated encephalitis had abnormal CSF findings, including 11 with abnormal cell counts, 1 with elevated levels of phosphorylated tau protein, and 3 with elevated levels of protein (**Table 1**). The CSF of the case patient had elevated leukocytes, and the percentage of lymphocytes was 85%. Then, various abnormal rheumatologic markers were assessed (**Table 1**). Autoimmune antibodies were present in some cases such as antinuclear antibodies, anti-dsDNA antibodies, anti-SSA/Ro52 antibodies, anti-Sm antibodies, et al. Herein, the patient had positive autoimmune antibodies but had no clinical manifestations such as recurrent fever, arthralgias, skin rashes, photoallergy, and mucosal ulcers. Moreover, cardiac ultrasound and chest CT scans did not reveal pericardial or pleural cavity effusion; thus, systemic lupus erythematosus could not be diagnosed. Nevertheless, close follow-up was required.

The confirmatory test for AE is positive anti-neuronal cell antibodies in the serum and/or CSF. The recommended methodology for detecting neuronal surface-antigen antibodies and some neurosynaptic intracellular antigen antibodies (e.g., GAD antibodies) is CBA. Other methodologies include protein blotting and TBA. Simultaneous testing for CSF and serum specimens is recommended (19). Because serum antineuronal antibody testing alone may lead to false-positive or false-negative results, CSF testing can improve the accuracy of the results (3). Of the 15 patients with neurexin-3a antibody-associated encephalitis reported to date, 12 were positive for both serum and CSF, 2 were seropositive only, and 1 lacked detailed information. The case patient’s blood and CSF samples were positive for neurexin-3a antibodies by CBA. TBA confirmed that both specimens were immunoreactive, exhibiting neuronal membrane antibody-positive fluorescence. Hence, the final diagnosis of neurexin-3a antibody-mediated encephalitis was confirmed.

Immunotherapy is the most effective treatment for AE according to the current guidelines; however, there is no consensus on the specific treatment for neurexin-3a antibody-mediated encephalitis. The current first-line agents used to treat AE are corticosteroids, plasma exchange, or intravenous immunoglobulin. When first-line interventions fail, second-line options include rituximab (CD20 receptor monoclonal antibody), cyclophosphamide (DNA alkylating agent), and other compounds, such as mycophenolate mofetil and azathioprine (20). Of the 15 patients with neurexin-3a antibody-mediated encephalitis reported thus far, 9 showed improvement in clinical manifestations, 2 showed no improvement, and 4 died, after treatment with steroid glucocorticosteroids, immunosuppression, or plasma exchange. The prognosis was poor in some cases, and even death occured. The potential reasons may be related to the severity of the patients’ clinical symptoms and poor efficacy of immunotherapy. According to literature reports, the clinical symptoms of some cases were similar to severe NMDAR encephalitis. Early intervention of first-line immunotherapy is very important.

In summary, neurexin-3a antibody-mediated encephalitis is very rare. It’s a new type of AE characterized by non-specific symptoms or mood changes in the early stage, followed by the rapid appearance of epilepsy, memory loss, confusion, loss of consciousness, central hypoventilation, behavioral abnormality, or speech disorders. Autoimmune antibodies were present in some cases and abnormal MRI signals were found in the brain of some patients. Some patients responded to immunotherapy, but the prognosis was poor in some cases, and even death occured. Therefore, when the patient has the above manifestations, clinicians should consider this disease and improve the relevant auxiliary examinations to clarify the diagnosis and guide the treatment, hence minimizing disability and saving the patient’s life.

## Data Availability

The original contributions presented in the study are included in the article/supplementary material. Further inquiries can be directed to the corresponding author.
